# A Retrospective Study of the Effects of Traumatic Brain Injury on Auditory Function: From a Clinical Perspective

**DOI:** 10.3390/neurosci3010004

**Published:** 2022-01-14

**Authors:** Mira White, Fauve Duquette-Laplante, Benoît Jutras, Caryn Bursch, Amineh Koravand

**Affiliations:** 1 Audiology and Speech Pathology Program, School of Rehabilitation Sciences, Faculty of Health Sciences, University of Ottawa, Ottawa, ON K1H 8M5, Canada; nrazr042@uottawa.ca (M.W.); fduqu103@uottawa.ca (F.D.-L.); 2 School of Speech-Language Pathology and Audiology, Faculty of Medicine, Université de Montréal, Montreal, QC H3N 1X7, Canada; benoit.jutras@umontreal.ca; 3 APD Ottawa, Audiology Private Practice, Ottawa, ON K2V 5G7, Canada; caryn@apdottawa.ca

**Keywords:** traumatic brain injury, audiology, auditory perceptual disorders, hearing tests, hearing disorders-diagnosis, auditory perception

## Abstract

Purpose: The main purpose of this retrospective study was to identify auditory dysfunctions related to traumatic brain injury (TBI) in individuals evaluated in an Audiology clinic. Method: Peripheral and central auditory evaluations were performed from March 2014 to June 2018 in 26 patients (14 males) with TBI. The age of the participants ranged from 9 to 59 years old (34.24 ± 15.21). Six participants had blast-related TBI and 20 had blunt force TBI. Sixteen experienced a single TBI event whereas ten experienced several. Correlation analyses were performed to verify the relationship, if any, between the number of auditory tests failed and the number, type, and severity of TBIs. Result: All participants failed at least one auditory test. Nearly 60% had abnormal results on degraded speech tests (compressed and echoed, filtered or in background noise) and 25% had a high frequency hearing loss. There was no statistically significant correlation between the number of auditory tests failed and the number, type, and severity of TBIs. Conclusion: Results indicated negative and heterogenous effects of TBI on peripheral and central auditory function and highlighted the need for a more extensive auditory assessment in individuals with TBI.

## 1. Introduction

Traumatic brain injury (TBI) refers to any traumatic damage to the brain from an external force that results in changes in cognitive and behavioral functioning [1]. Injury to the brain is typically defined by the manifestation of the following clinical symptoms: any period of loss or decreased level of consciousness, memory loss immediately before or after the injury, any alteration in mental state at the time of injury (feeling dazed, confused, disoriented, etc.), neurological deficits (such as paresis/plegia, aphasia, sensory loss, etc.) and intracranial lesion [1,2,3,4]. The severity of the injury could be classified as mild, moderate, or severe, based on the patient’s Glasgow Coma Scale (GCS) [5,6]. The external forces that cause TBI include non-blast related forces, such as “the head being struck by an object, the head striking an object, acceleration/deceleration movement of the brain and penetration of foreign body into the brain” [4] (p. 288), and blast-related forces that can be generated from events such as a blast or explosion [7]. In the present study, the external forces are divided into two categories: blast-related trauma and blunt force trauma. Blast-related trauma is often a consequence of the use of explosive devices in military operations [7,8,9]. The high energy explosion created from the extremely rapid conversion of a solid or liquid into gases causes a sudden increase in pressure in the surrounding atmosphere. The gases expand swiftly, decompressing the surrounding air and creating a supersonic over-pressure wave or positive pressure [9,10]. As the gases expand in all directions, a pressure drop occurs, resulting in negative pressure or an under-pressure wave [10]. The extreme pressure differences could cause stress and shear forces on tissues of the human body, which are referred to as primary blast force injuries [9,10]. The body and the brain are vulnerable to blast injuries, although there are gaps in knowledge regarding the exact transduction pathway in the brain. Changes to the pressure of the blood vessels in the brain, transcranial propagation, and pressure changes through the spinal cord or fluid are only three of the many ways a blast force trauma can cause injuries to the brain [11]. Blast force also affects air-fluid interspaces in the human body, including lungs, bowels, and middle ear, and as such, damage to tympanic membranes may occur, which is referred to as barotrauma of the ear [12]. Due to exposure to loud sounds stemming from explosives, noise-induced hearing loss may also occur. However, contrary to the sudden impact on the integrity of the auditory system from extreme pressure differences, noise-induced hearing losses develop slowly over many years [13]. Additionally, noise-induced hearing loss is sensorineural in nature, mainly affecting the hair cells of the inner ear [14]. Any auditory dysfunction following a blast force injury could be due to tympanic membrane rupture, loss of hair cells in the inner ear and/or due to neurological injury [14,15], all of which need to be addressed by the healthcare professional treating the patient.

TBI stemming from blunt force, on the other hand, is a result of direct impact of an object with the head, resulting in changes to the integrity of the brain [16,17]. This includes falls, motor vehicle accidents and direct assaults [18]. As a result of the impact, the blood–brain barrier is compromised, destroying the neuronal and glial tissues, causing local inflammation and secondary neurodegeneration [19]. Consequently, TBI can cause many intracranial disturbances, such as subdural and epidural hematomas (injury resulting in mass lesions), cerebral contusions (the brain shaking inside the skull due to acceleration-deceleration force), diffuse axonal injury (rapid deceleration force causing tearing and shearing of neurons), subarachnoid hemorrhage (lacerations in the pial blood vessels), and intracerebral hematoma or bleeding (provoked by an amalgam of contusions or a tear in a parenchymal vessel) [20]. In more severe brain injury cases that occur with temporal bone fracture, disruption of the structure of the middle ear and/or inner ear sensory neuroepithelium is considered as the direct cause of hearing loss [21,22]. Without the temporal bone fracture, it can be more difficult to predict the outcome on the patient’s hearing [23]. The difference between damage caused by blast force trauma and blunt force trauma has not yet been fully identified, as patients with either injury experience the same pathologies: diffuse axonal injury, contusions, hemorrhage, and hematomas [19,20,23]. However, in some cases, blast force trauma is further complicated by toxic inhalation and radiation exposure [19].

In Canada, self-reported TBI increased by 1.4% annually between 2005 and 2014 [24]. During 2014, it was estimated that about 155,000 people suffered from a TBI [24]. According to Roebuck-Spencer and Cernich [25], the number of affected individuals could be higher, since those experiencing mild symptoms may not have sought medical help. In addition, Oleksiak et al. [26] found that 65.1% of the veterans with mild TBI, who complained of hearing loss were not provided with a referral, even though studies have revealed the negative effects of TBI on hearing [27,28,29,30,31,32]. Studies performed in veterans following explosive injuries have shown damage to the structures of the peripheral hearing system (outer, middle and/or inner ear) which has resulted in hearing loss and tinnitus [33,34]. However, some veterans with TBI showed clinically normal hearing thresholds, but performed below average on tests assessing the central auditory system [35,36,37]. A dysfunction of the central auditory system was also revealed by abnormal fundamental frequency processing and neural responses in children, adults, and athletes with a history of TBI [30,38,39], as well as listening-in-noise difficulties in children with TBI [31,40]. In a case study report performed by Fligor, Cox and Nesathurai [41], a 30-year-old woman had presented with clinically normal hearing thresholds 13 years post-injury but was found to have abnormally functioning neurogenic potentials in the injured ear, based on the results of brainstem auditory evoked response tests. This dysfunction could be repercussions from the exaggerated back and forth movement of the brain within the skull during the TBI. These movements can cause shearing or stretching of the axons and small vessels, resulting in the improper signaling of cells, which in turn may lead to axonal death or disconnection [11,42]. Furthermore, previous studies have shown the type of trauma, higher number of brain injuries, as well as severity of injury may play a role in the severity of symptoms suffered by patients [43,44,45]. This will be explored in the current study.

The aim of the current study was to investigate the effects of TBI on auditory function in a cohort of patients who visited an audiology clinic from March 2014 to June 2018. More specifically, the objectives of the current study were to explore (1) any difficulties with their peripheral and central auditory systems following blast-related force and blunt force TBIs; and (2) the relationship between the type, number, and severity of TBI and auditory difficulties.

## 2. Materials and Methods

All procedures were approved by the Office of Research Ethics and Integrity at the University of Ottawa (Ethics File Number: H-10-18-1217). The experiment was designed as a retrospective cohort study.

### 2.1. Participants

All audiological case files of patients who visited an audiology clinic in Ottawa from March 2014 to June 2018 were reviewed to identify those with a TBI. From this review, 26 patients (14 males), aged between 9 to 59 years old (mean 34.24; standard deviation 15.21) and noted to have had a TBI, were included in this study. For most of the participants, the diagnosis of a positive TBI, including its severity, was provided by either a family physician, an emergency room doctor, or a neuropsychologist. Three patients could not access care at the time of the injury and were diagnosed later by their family physicians. Nineteen patients visited the audiology clinic on their own because they were concerned about their hearing symptoms, while the other seven patients were referred to the clinic by a physician. Six patients reported having had blast-related TBI and 20 had blunt force TBI. Those with blast-related TBIs were military veterans who had prior exposure to proximate improvised explosive device explosions, demolitions, firing in close range to anti-aircraft guns and hazardous levels of noise from aircrafts. Ten participants with blunt force TBIs experienced motor vehicle accidents, three had falls and seven experienced assaults (including those from contact sports). Additionally, among the participants with blunt force trauma, nine had whiplash head injury, five had experienced a blow to the back of the head, three had experienced a hit to the side of the head, one participant had been hit on the very top of the head (compression component to the neck) and one had been hit on the forehead. Information regarding the cause of injury was absent in the report of one participant. This participant was classified in the blunt force TBI group based on the information in the file about his work and leisure activities. Furthermore, the degree of severity was not available for three participants who experienced blast-related trauma, while three reported moderate TBIs. Sixteen participants had a single TBI event and 10 had several TBI incidents. Twenty-one participants reported no prior hearing issues, three had multiple ear infections as a child and information on two of the participants was missing. (see Table A1, Appendix A).

### 2.2. Data Collection

Collected data were related to age, sex, reason for visit and history of concussion or head injury. Moreover, auditory tests assessing peripheral and central function were also compiled from the client files. These tests were administered by two audiologists with, respectively, four and thirty years of clinical experience who diagnosed/evaluated clients according to the Canadian Guidelines on Auditory Processing Disorder in Children and Adults: Assessment and Intervention [46]. Data from peripheral hearing tests, such as otoscopy, tympanometry, pure tone air and bone audiometry, distortion product otoacoustic emissions, and word recognition in quiet (NU#6 recorded lists Ordered by Difficulty) [47] were analyzed, and patients with hearing loss were identified. Otoacoustic emissions (OAEs) were measured from 500 Hz to 8000 Hz. They were described as absent when there was no response obtained at all frequencies tested. Partially absent OAEs were defined as a measurable threshold for at least one of the frequencies tested, but not at all frequencies. For adult participants (18 years old and older), hearing sensitivity was qualified as being within normal limits when thresholds were at 25 dB HL or less. For participants younger than 18 years of age, the normal range was defined as thresholds at 20 dB HL or less, for frequencies between 250–8000 Hz. Hearing loss was defined as thresholds above the aforementioned criteria and was labeled high frequency hearing loss when the thresholds were abnormal only at 6000 Hz and/or 8000 Hz.

Regarding the assessment of the higher part of the auditory system, central auditory abilities were measured with specific tests. For example, auditory attention was tested with the Auditory Continuous Performance Test (ACPT) [48,49]. Auditory temporal abilities were evaluated with the Random Gap Detection Test [50], the Frequency Patterns Test (FPT) and the Duration Patterns Test (DPT) [51,52,53]. Information for auditory closure was obtained from the Time-Compressed Speech Test (TCS) [54,55] and the Filtered Words (FW) Test with a low-pass filter at 1000 Hz [56]. Tests for speech in noise included Listening in Spatialized Noise (LiSN-S) [57,58], Quick Speech in Noise (QuickSIN) [59,60] and the Bamford-Kowal-Bench Speech in Noise Test (BKB-SIN) [61,62]. Binaural separation was evaluated with the Competing Sentences Test (CST) [63,64], and binaural integration data were drawn from the Staggered Spondaic Words test (SSW) [65,66] and the Dichotic Digits test (DD) [67,68]. A minimum of one test for each auditory function was administered. Participants’ test results were compared with age-matched normative data. Participants diagnosed with misophonia, a type of hearing disorder that causes physiological and emotional responses upon hearing certain sounds [69], were classified into a separate category. Participants who were not diagnosed with misophonia, but experiencing negative physical reactions, such as anger, pain, or fear, to certain sounds or at certain frequencies were placed into the category “negative physical reactions to sounds”. Data from the audiological files were anonymized and stored in a secure electronic database (Excel, Microsoft™).

### 2.3. Statistical Analysis

All statistical analyses were performed using SPSS (IBM Corp. 2017, Version 25.0, RRID:SCR_019096). To explore the relationship between the degree of auditory dysfunction (i.e., number of failed tests) and the number of TBIs or the number of years that have passed since the most severe TBI occurrence, Pearson product-moment correlation was used, with a *p* < 0.05 for significance level. Parametric test was used to test these variables because the variables are continuous in nature. Furthermore, the Kruskal–Wallis non-parametric test explored the effect of the degree of TBI severity on the number of auditory tests failed (significance level of *p* < 0.05). A non-parametric test was used to test these variables because one of the variables is discrete in nature, while the other is continuous. Finally, binary logistic regressions calculated the risks of having auditory dysfunction according to the type of trauma experienced (blunt- or blast-related force trauma) or to the number of TBIs (one vs. several). A binary non-parametric test was used to test these variables because both variables are discreet and binary in nature.

## 3. Results

### 3.1. Auditory Dysfunction in TBI Participants

**Figure 1:**
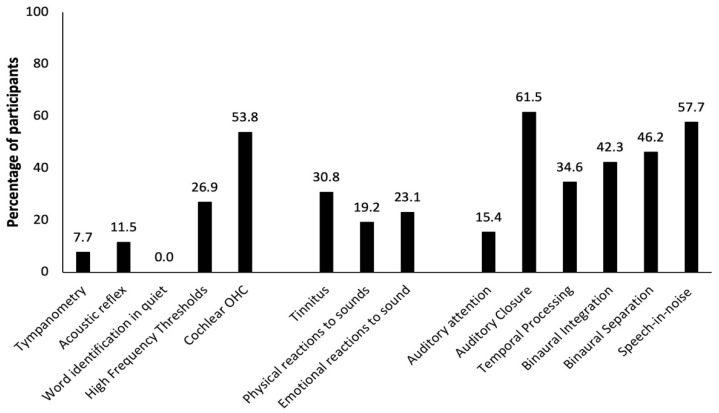
Percentage of TBI participants with a particular auditory dysfunction.

Results showed abnormalities in the peripheral and central auditory system among the participants with TBIs (Figure 1). Peripheral auditory dysfunction was highlighted by partially absent or absent otoacoustic emissions (abnormal cochlear outer hair cell function) in more than 50% of the 26 participants with TBI. A quarter of them had high-frequency hearing loss. Results of the other peripheral auditory tests—tympanometry, acoustic reflexes, and word identification in quiet—were within the normal limits for most of the participants. Close to one third of the participants reported having tinnitus, whereas almost a quarter of the participants revealed having emotional reaction to sounds (misophonia) and a fifth reported a negative physical reaction to sounds. With regard to the function of the central auditory system, auditory dysfunction was present in many participants. Indeed, approximately 60% of the participants obtained abnormal results on tests assessing auditory closure and speech in noise perception. Binaural integration and separation tests were failed by more than two-fifths of the participants and auditory temporal processing tests by more than a third. The auditory attention test was failed by only 15% of the participants.

### 3.2. Relation between TBIs and Auditory Dysfunction

Calculation of a Pearson product-moment correlation coefficient revealed that there was no significant relationship between the number of tests failed and the number of TBIs (r = 0.146, *n* = 26, *p* = 0.478) or the number of years since the occurrence of the most severe TBI (r = 0.131, *n* = 22, *p* = 0.56). Results of the Kruskal–Wallis test showed no significant relationship between the number of auditory tests failed and the degree of the TBI severity [H (2) = 0.067, *p* = 0.967]. Additionally, the likelihood of failing any of the auditory tests (including central processing tests) was not significantly different between participants with one versus several TBIs, or between blast force TBI versus blunt force TBI (Table 1). However, having abnormal cochlear outer hair cell function (measured with otoacoustics emissions) or difficulties with listening in noise was almost 5 times more likely to occur in participants with several TBIs than those with one TBI (*p* = 0.08) (Table 1).

## 4. Discussion

One objective of this study was to explore auditory dysfunction in the peripheral and central auditory systems secondary to a TBI. The results of the current study revealed that most of the participants with TBI had some degree of peripheral and central auditory dysfunction. More than 50% of the participants had abnormal otoacoustic emissions, and experienced difficulties with word identification in noise, as well as with auditory closure. Difficulties with word identification in noise is in concordance with previous studies that have found substantial numbers of participants with TBI experiencing difficulty listening in noise [29,37,70]. Difficulties with auditory closure were also found in previous studies showing the impact of a TBI on this auditory ability [15,35,36]. These results may support the necessity to include tests of auditory closure, such as time-compressed speech and speech in noise in the clinical test battery in order to shed light on potential difficulties with degraded speech following TBI. The current study also showed that out of the nineteen participants who had otoacoustic emissions tests, 67% had abnormal otoacoustic emissions around 3000–4500 Hz. The risk of abnormal otoacoustic emissions is usually related to the presence of hearing loss. In the present study, of the 15 participants having abnormal otoacoustic emissions, the majority (67%) had normal pure tone thresholds. Abnormal results on central auditory tests and otoacoustic emissions in combination with normal hearing thresholds might lead one to believe that TBI may cause hidden hearing loss in some individuals. Supported by an animal model, Monaghan et al. [71] showed that exposure to noise, even temporarily, can affect the neural coding of speech along the auditory fibers for speech in background noise, even with normal hearing thresholds. As documented in their review, Kohrman et al. [72] reported several causes of hidden hearing loss other than noise exposure, such as age, auditory neuropathy, and ototoxicity. Based on the present study, TBI might be another cause of this type of hearing loss. This must be investigated further.

The results of the present study also showed no correlation between the number of TBIs and the amount of auditory dysfunction. These findings are consistent with those of Bryan [73], who tested military personnel with a single, several and no TBIs. No significant difference in hearing disorders was found between those with a single TBI and those with multiple TBIs. Moreover, the degree of TBI severity seemed not to influence the number of failed auditory tests. The data revealed that participants with mild TBI, on average, failed the same number of tests as those with a moderate or severe TBI. These findings suggest that few or more auditory difficulties may be present in individuals with mild TBI as well as in those with moderate or severe TBI. In addition, the results of the present study did not show a significant relationship between type of auditory dysfunction and type of TBI. This is in line with the results of Bryden et al. [74], Das et al. [19], Greer et al. [75] and Lubner et al. [23]. They showed very little or no difference in the auditory test outcomes between the participants suffering from blast-related force trauma and those having blunt force trauma. However, results from other studies revealed a greater incidence of auditory dysfunction among blast force trauma patients compared to blunt force trauma patients [76,77,78]. The authors claimed that blast-related force would induce more severe auditory impairments due to the more global brain injury [77]. Blast-related force would also likely cause some changes over time in neurochemicals and gene expression [77] in addition to inducing neuronal injuries, including expanded perineuronal spaces, cytoplasmic vacuoles, myelin deformation and axoplasmic shrinkage in the hippocampus and brainstem reticular formation areas [79]. Blunt force trauma would only impact a confined zone of injuries, resulting in localized axonal damage [77]. The study by Hoffer et al. [77] found worse hearing loss among participants with blast injuries, with additional impacts on the integrity of the vestibular system. The integrity of the vestibular system was not tested in the present study. Additionally, the study only tested pure-tone audiometry to obtain information on their hearing loss, which is arguably insufficient considering the lack of information on central auditory processing function. Additionally, the study recruited participants with pure blunt and pure blast-related injuries, which the present study lacks. In the study performed by Belanger et al. [76], the degree of injuries of the participants was measured using questionnaires or were self-reported. It is very likely that the participants with blast-related force trauma in the current study perceived a greater degree of hearing difficulty than the blunt force trauma group, but this was not further explored. Furthermore, the author had attributed the difference in perceived hearing difficulty between the two groups of participants to emotional distress and time since injury.

## 5. Study Limitations

Due to the nature of this retrospective study, the performed audiology test battery was not uniform across the participants and some data were missing. The current investigation also had limited information regarding the nature of the blast force trauma injuries suffered by the participants, such as whether they were pure or mixed injury, the distance of the participants from the explosives, and details of the surrounding environment. Thus, prospective studies need to take these factors into consideration. Another limitation of this study was the size of the sample, the unequal number of participants with each type of TBI and a lack of information on the hearing status prior to the TBI. The heterogeneity of the participant population also makes the results difficult to interpret. Specifically, the wide range of age and TBI characteristics can be of concern. The type of injuries varies greatly among the participants, and those with blast-related force trauma may have experienced blunt force trauma previously, or as a secondary or tertiary injury. Finally, information regarding the site of injuries using imaging technology for assessing affected specific axonal tracts would have been useful for understanding, in depth, the effects of brain injury on the auditory system. However, the imaging results were not acquired or were unavailable.

## 6. Conclusions

Results of the current retrospective study of participants with TBI revealed that the majority of participants experienced auditory dysfunction in the peripheral and central auditory systems, regardless of the number, type, and severity of the TBI(s). The present study highlighted the specific impacts of the TBI on auditory function and therefore, the importance of receiving a full audiological assessment post TBI. This is essential for planning interventions aimed at returning to daily living activities, such as work, school, and leisure. Interventions could be tailored for each patient according to the specific auditory dysfunction, thereby increasing the chances of improving the quality of life for patients after a brain injury.

## Figures and Tables

**Table 1:** Odds ratio (OR) of auditory dysfunction experienced by participants with several TBIs compared to those with only one TBI or with blast-related force TBI compared to those with blunt TBI. The 95% confidence interval (CI) and *p*-values (*p*) are listed.

	Several vs. One TBI			Blast vs. Blunt Force TBI		
Auditory Dysfunction	OR	CI	*p*	OR	CI	*p*
Cochlear outer hair cells function	5.143	0.819–32.303	0.081	8	0.12–523.32	0.530
Tinnitus	4.333	0.742–25.295	0.103	3	0.4516–19.929	0.256
Physical reactions to sounds	0.389	0.0372–4.061	0.430	0.750	0.067–8.382	0.815
Misophonia	0.244	0.024–2.489	0.234	0.600	0.056–6.442	0.673
Auditory Closure	0.455	0.089–2.318	0.343	1.333	0.196–9.083	0.769
Temporal processing	1.467	0.282–7.627	0.649	2.333	0.362–15.054	0.373
Auditory Attention	0.482	0.043–5.401	0.554	1.133	0.097–13.441	0.921
Binaural Integration	0.857	0.172–4.267	0.851	1.500	0.239–9.383	0.664
Binaural Separation	1.286	0.264–6.276	0.756	1.222	0.197–7.595	0.829
Speech-in-noise	5.143	0.819–32.302	0.081	5.000	0.492–50.833	0.174

## Data Availability

The data presented in this study are available on request from the corresponding author. The data are not publicly available due to participant privacy.

## Appendix A

**Table A1:** Specifics on participants’ injuries, as well as test results.

Analysis	Sub-Categories	No. of TBIs Sustained		Type of TBIs Sustained	
		Single (*n* = 16)	Multiple (*n* = 10)	Blunt Force (*n* = 20)	Blast Force (*n* = 6)
**Reason for visit**	Physician’s referral	4	3	5	5
	Hearing difficulties	12	7	15	1
**Auditory issues prior to TBI**	Yes (middle ear infections as a child)	-	3	3	-
	No	15	6	16	5
	Unknown	1	1	1	1
**Type of Injury**	Explosions	2	4	-	6
	Motor Vehicle Accidents (MVA)	6	3	9	-
	Falls	4	-	4	-
	Assault and contact sports	2	2	4	-
	Unknown	2	-	2	-
**Degree of Severity (for multiple TBIs, the most severe injury is listed)**	Mild	7	-	7	-
	Moderate	4	2	4	2
	Severe	1	3	3	1
	Unknown	4	5	6	3
**Specifics of Injury (on blunt force trauma)**	Whiplash	-	-	9	-
	Blow to the back of the head	-	-	5	-
	Blow to the side of the head	-	-	3	-
	Hit from the top of the head (neck compression component)	-	-	1	-
	Hit on the forehead	-	-	1	-
	Unknown	-	-	1	-
**Time since injury (time since the most severe injury for multiple TBIs)**	1 year or less	4	-	4	-
	Between 2 to 5 years	4	-	4	-
	6 years or more	6	8	9	5
	Unknown	2	2	3	1
**Cognition pre-injury**	Learning Impairment	3	1	2	1
	Unknown	1	5	6	2
	Excellent	12	4	12	3
**Cognition post-injury**	Slow processing speed	8	3	9	2
	Disoriented	1	2	1	1
	Difficulty concentrating	7	5	7	4
	Some memory issues	4	2	6	-
	Unknown	3	3	7	2
**Other sensory disturbances**	Vision/Light sensitivity	10	4	13	2
	Smell	3	-	3	1
	None	-	2	1	3
	Unknown	4	1	4	-
**Otoscopy Results**	Normal both ears	15	9	19	5
	Abnormal both ears	1	-	1	-
	Unknown	-	1	-	1
**Tympanometry**	Within normal limits	12	5	16	1
	Abnormal	1	1	2	-
	Unknown	3	4	2	5
**Acoustic Reflex**	Normal both ears	9	4	12	1
	Elevated or absent in either ear	1	2	3	-
	Unknown	6	4	5	5
**Cochlear Hair Cells Functions (DPOAE)**	Normal	3	1	4	-
	Abnormal	7	8	10	5
	Unknown	6	1	6	1
